# Review of Biopolymer Polyhydroxybutyrate (PHB) and Blends: Modification of Thermal and Mechanical Properties via Additive Manufacturing Processing

**DOI:** 10.3390/polym17223083

**Published:** 2025-11-20

**Authors:** Dan Li, Yunxia Yang, Ruochen Liu, Yufeng Wu, Fu Guo

**Affiliations:** 1 College of Materials Science and Engineering, Beijing University of Technology, Beijing 100124, China; lidan@bjut.edu.cn (D.L.);; 2 Key Laboratory of Advanced Functional Materials, Education Ministry of China, Beijing University of Technology, Beijing 100124, China; 3 School of Mechanical Engineering and Automation, Beihang University, Beijing 102206, China; 4 Institute Circular Economy, Beijing University of Technology, Beijing 100124, China; 5 School of of Mechanical Electrical Engineering, Beijing Information Science and Technology University, Beijing 100192, China

**Keywords:** polyhydroxybutyrate, poly-3-hydroxybutyrate P(3HB), poly-4-hydroxybutyrate P(4HB), polymer blends, material processing

## Abstract

The non-degradable polymers used in daily and commercial application are generally inexpensive; however, their excessive use leads to extensive environmental damage. In light of this, the demand for bio-derived, biocompatible, and biodegradable polymers increases since these materials are potential alternatives to petroleum-derived polymers. Polyhydroxybutyrate (PHB), a class of highly crystalline thermoplastics derived from natural sources, offer significant environmental advantages over fossil fuel-based polymers due to their inherent biodegradability. This eco-friendly profile has spurred research into their commercial applications, ranging from food packaging to pharmaceuticals. However, processing challenges, particularly for polyhydroxybutyrate (PHB)—including high costs and the requirement for elevated temperatures—remain major obstacles. Additionally, PHB-based products are often brittle and exhibit inferior mechanical properties compared to conventional petroleum-based polymers such as polypropylene and polyethylene. This review comprehensively examines the state-of-the-art processing techniques for PHB and their composites. Key properties, such as mechanical performance, thermal behavior, and degradation characteristics, are scrutinized. Furthermore, the review explores mitigation strategies, such as blending and plasticization, aimed at overcoming the mechanical brittleness while upholding the principles of sustainability and maintaining a low carbon footprint.

## 1. Introduction

Polymers encompass not only the synthetic plastic bottles and bags, but also textiles and natural polymers that are important components of life, such as DNA and proteins [1]. Natural and synthetic polymers are both extensively used in our daily life. The packaging materials used currently are dominated by fossil fuel-based plastics, including polyethylene (PE), polypropylene (PP), and polyethylene terephthalate (PET), leading to plastic waste accumulation [2,3]. Beyond the environmental issues caused by large plastic objects, there is growing evidence of microplastics and their associated chemicals accumulating in the marine environment, food chain, and drinking water—the consequences of exposure are horrifying [4,5]. The demand for biologically derived, biodegradable polymers is increasing since they are environmentally attractive alternatives to fossil-fuel-based polymers [6,7]. The bioplastic market is estimated to expand to almost triple from 2022 to 2027, up to 6.3 Mt globally [8,9]. Among the bio-derived and biodegradable plastics, polyhydroxyalkanoate (PHA) is particularly being investigated owing to its rapid biodegradability in nature [10,11,12].

PHAs are synthesized by microorganisms through a process that involves the fermentation of sugars, lipids, or other carbon sources. The most common PHA, polyhydroxybutyrate (PHB), is produced by about 40 to 60 species of microorganisms according to various sources. The biopolymers PHB, poly-3-hydroxybutyrate P(3HB), and poly-4-hydroxybutyrate P(4HB) are bio-based and biocompatible, possessing significant advantages over fossil-fuel-based polymers with regard to environmental impact. PHB is a short-chain-length PHA with a highly crystalline structure, which gives it excellent mechanical properties but also makes it brittle. P3HB is a highly crystalline material, while the reduction in P4HB crystallinity enhances its processability and mechanical ductility. P4HB’s unique position has led to it being the only member of the PHA family to obtain FDA approval for medical applications, a status held since 2000. Since 2007, the FDA has approved, for use in medicine, products made from P4HB by Tepha (a subsidiary of Metabolix), manufactured under the TephaFLEX trademark, as well as bioabsorbable suture material (manufactured by Bayer), mesh endoprostheses, and endoprostheses for tissue plastic surgery [13,14,15,16,17].

PHB can be produced from a variety of renewable resources, including agricultural waste, food waste, and even carbon dioxide (CO_2_), through microbial fermentation. The use of waste materials as feedstocks not only reduces the cost of PHB production but also contributes to a circular economy by converting waste into valuable products [18]. Recent advances in metabolic engineering and synthetic biology have further optimized the production processes, enabling higher yields and lower costs. PHB is produced via biological fermentation, during which it accumulates as intracellular granules that serve as energy reserves. These granules are harvested post-fermentation, yielding a high molar mass polymer (up to 1000 kDa). To obtain lower molar mass formulations, the material must undergo chemical hydrolysis, an additional post-fermentation step that increases both process complexity and production costs. Although a key advantage of this fermentation-based route is the absence of residual metal catalysts, the high overall cost of bio-P4HB, driven by slow reaction kinetics and limited production scale, currently hinders its practical large-scale implementation [19].

There are three different classes of properties that are considered when assessing the potential of a polymer for a given application: (1) intrinsic properties related to the polymer structure, further splitting into molecular and bulk difference, (2) processing properties during formation (e.g., viscosity and melt flow index), and (3) final product properties. Hence, this review focuses on the processing of PHB and its blends, the use of commercially scalable processing methods, and the environmental impact of PHB and its blends, analyzed using LCAs summarized from data in recent years. The objectives of this review are to:

1. Evaluate a rational design of bioplastics’ properties for real use (e.g., packaging).
2. Discuss PHB and bio-blends/plasticizers and their potential to enhance the mechanical properties of bio-composites.
3. Investigate PHB and blends using the commercially scalable processes, including hot-processing, chemical melt extrusion, and additive manufacturing techniques.
4. Compare the carbon footprint and environmental impacts of PHB bioplastics with varying recycling strategies.

## 2. Background

### 2.1. General Aspects About Bioplastics

The global demand for bioplastics is rising rapidly due to increasing environmental concerns. While traditional petrochemical plastics, like polyethylene (PE), are widely used for their durability, barrier properties, and low cost, they pose serious environmental risks due to their poor degradability. Similarly, not all bioplastics are inherently biodegradable or recyclable, especially single-use items that often end up in landfills, threatening ecosystems and human health [6].

Biopolymers are generally defined into three genres:

Type A: both biodegradable and bio-based

Type B: not biodegradable but bio-based

Type C: biodegradable but made from fossil fuels

**Figure 1:**
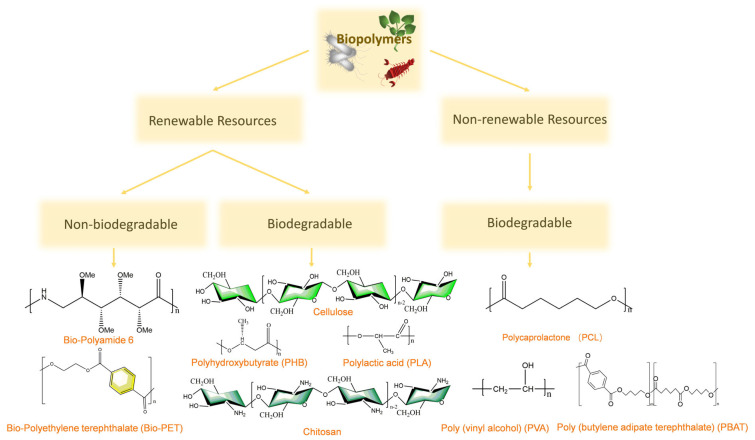
Different types of biopolymers (bioplastics).

Figure 1 displays the plastic coordinate system, which separates plastics in categories based on the source of its production and on whether it is biodegradable or not. Biopolymers are separated into two categories and are highlighted into three portions in Figure 1. From the top to the bottom of the schematic in Figure 1, biopolymers are divided into two sections based on their source: derived from renewable or non-renewable resources. The renewable section has been further divided into two sections: non-biodegradable and biodegradable, and the same applies to the non-renewable section. For example, poly (caprolactone) (PCL), a biopolymer used in the biomedical field, is produced from fossil fuels, but it is biodegradable in mild conditions. In general, the ideal biopolymer to consider is one derived from renewable resources and can be biodegraded easily, for instance, polyhydroxyalkanoates.

### 2.2. Structure and Properties of PHB and Its Copolymers

#### 2.2.1. Biopolymers

PHAs are ecofriendly and sustainable plastics that are compostable in all mediums. The U.S. Food and Drug Administration (FDA) has approved PHB for food-contact uses, whereas PHAs are certified as completely bio-derived biopolymers that are biodegradable and compostable in an ambient environment at a relatively rapid speed.

**Figure 2:**
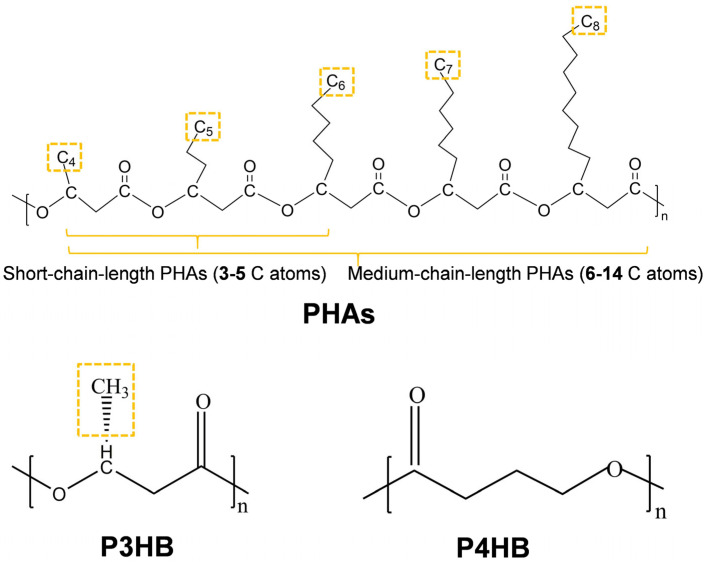
PHAs, P3HB, and P4HB chemical structure.

PHB, a highly crystalline thermoplastic in the polyhydroxyalkanoate (PHAs) aliphatic polyester class, was first isolated and characterized by Lemoigne et al. in 1927 [20]. P3HB and P4HB are produced by many types of bacteria, with the more common form being P3HB; their monomer, R-3-hydroxybutyric acid, is non-toxic and can be completely catabolized. Figure 2 presents the general structure of PHAs, in which the difference in v properties in this PHA family being determined by the R- substituent bonded to the main polymer chain [21]. The R-substituent is a methyl group (i.e., -CH_3_) in the case of PHB, which is the more commonly used material from the PHA family. Short-chain-length PHAs have three to five carbon atoms, including P3HB, P3HV, P4HB, and their copolymers; six to fourteen carbon atoms are considered medium-chain-length PHAs, such as P3HD, P3HO, and P3HTd. Medium-chain-length PHAs exhibit better elasticity compared to short-chain-length PHAs. Thus, blending different polymers may yield the desired mechanical strength and ductility in polymer products, which may also have an improved thermal stability.

#### 2.2.2. Polyhydroxybutytrate (PHB)

##### PHB Production

PHA production incurs high costs due to the expensive fermentation facilities required despite there being many large-scale production facilities around the world. The scaling up of PHA biosynthesis processes began in the late 1980s with extensive experience accumulating over the past 35 years. For biological synthesis, the choice of bacterium, its PHA polymerase (PhaC), and the carbon source allow for the production of PHAs with tailored monomeric compositions and mechanical properties. These microbial producers yield high-molecular-weight polymers (>10^6^ g mol^−1^), whose values can be fine-tuned by adjusting PhaC activity and concentration. A key benefit of this biological route is its avoidance of the toxic metal catalysts (e.g., organic tin compounds) typically required in chemical polyester synthesis [22,23]. Biomass uses carbohydrate materials as essential fermentation substrates, incorporating forestry and agricultural wastes. Additionally, food waste and other residues can also be utilized in the process. Lignocellulosic biomass is considered a prime carbon source for PHA production due to its abundant natural availability and high polysaccharide content [24]. This approach is often supplemented with a carbon source/feedstock commonly derived from agricultural products. The hydrolysates of various agricultural crops, such as rice husks, vegetative parts of wheat, Jerusalem artichoke, nut shells, and the pulp of various fruits, are used as substrates [25].

##### PHB Properties

The properties of P(3HB) and P(4HB) are influenced by their chemical structure and molecular weight. These varieties of P(3HB) and P(4HB) differ not only in the length of pendant chains but also in the number of carbon atoms in the main chain. Their thermal stability and mechanical properties can be enhanced through physical blending with natural materials and other biopolymers, such as cellulose, PLA, and starch, or through chemical modifications like block copolymerization and graft copolymerization. The general thermal and mechanical property data are listed in Table 1 [26,27,28,29,30,31].

**Figure 3:**
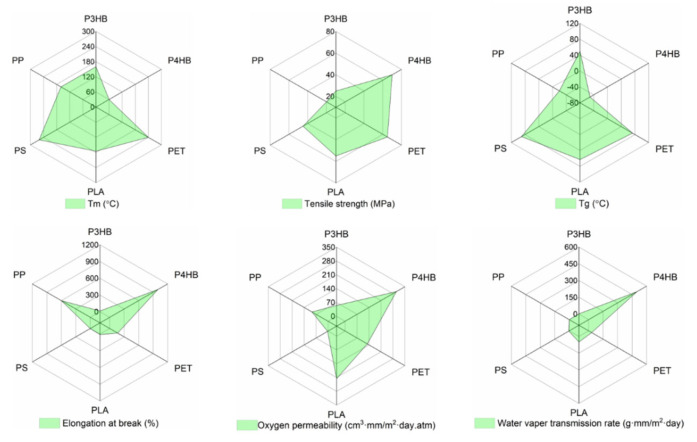
The typical properties (Tm, tensile strength, Tg, elongation at break, oxygen permeability, and water vapor transmission rate) of conventional plastics (PP, PET, PS, PLA) vs. P3HB and P4HB.

Given these properties, PHBs are regarded as promising alternatives to fossil fuel-based plastics but currently with very limited applications due to their thermal and mechanical property deficiencies. PHB, a commercial PHA prototype, is brittle, stiff, and has limited ductility (2–15% strain, >35 MPa yield stress) due to its high crystallinity (60–80%). Although PHB has comparable mechanical properties to polypropylene (PP), the thermal processing window of P3HB is quite limited for practical applications. The melting point is different from the PHB degradation temperature, while the processing temperature being close to the melting temperature may render the thermal aging of the PHB. Therefore, copolymerization of short-chain PHB is a viable approach to build a robust, flexible, and durable alternative with enlarged thermal processing capabilities. PHBV is a copolymerization of P3HB and PHV, showing relatively robust, strong, and enhanced thermo-mechanical properties. Compared to P3HB, P4HB is a strong, thermoplastic material that exhibits significantly greater flexibility than synthetic resorbable polymers, like poly-L-lactide (PLLA). It possesses a tensile strength comparable to ultra-high-molecular-weight polyethylene and an elongation at break of approximately 1000%. A key advantage of P4HB is its ability to retain flexibility even when its mechanical strength increases upon stretching—a behavior that contrasts sharply with PLLA, which becomes more brittle as its strength increases. The mechanical properties are further influenced by processing history; for instance, the strength of P4HB is greatly enhanced by fiber orientation achieved through melt-spinning, and these fibers can be further processed via braiding, knitting, or weaving (The processing of which will be extensively discussed in Section 4). Figure 3 gives the typical properties (tensile strength, elongation at break, Tg, and Tm) of conventional plastics and PHB [32,33].

## 3. Predictive Models and Future Perspectives on PHB Degradation

Degradation is a process by which polymer chains are broken down into smaller chains, resulting in varied physical characteristics of the polymer and produced degradation products (e.g., monomers and oligomers) [34,35]. The degradation behavior and physical properties of biopolymers are affected by factors including the structure of polymer backbone, synthesis approach, processing parameters, and degradation processes [36].

The biodegradability of blended polyhydroxyalkanoates (PHAs) is a complex yet critical research area, influenced by multiple factors including the physicochemical properties of the material itself, environmental conditions, and microbial communities. Key material properties such as crystallinity, monomer composition, molecular weight, and surface morphology significantly affect degradation rates. For instance, copolymers with a lower crystallinity (e.g., PHBV) generally degrade more readily than highly crystalline homopolymers (e.g., PHB) [37]. Additionally, environmental factors such as temperature, oxygen availability, pH, salinity, and humidity play important roles in the degradation process. In marine environments, factors such as temperature, light exposure, and salinity influence degradation rates, with higher degradation observed during summer due to elevated temperatures and increased microbial activity. In freshwater environments, PHB degradation proceeds more slowly, often taking months or even years, depending on the material’s properties and environmental conditions [38]. Although existing studies indicate that blended PHA exhibits good biodegradability, many issues remain to be thoroughly explored.

P3HB and P4HB undergo degradation through both non-enzymatic and enzymatic hydrolysis within the human body. This process breaks down the polymers into metabolites that are naturally occurring in body, which accounts for their favorable biocompatibility and suitability for medical applications [39]. The hydrolysis of P4HB in vivo is initiated by the diffusion of water into the polymer matrix, leading to chain scission. Enzymatic activity, potentially from non-specific esterases and lipases, then drives surface erosion of the polymer [40,41]. As a result, P4HB-based medical devices, like PHASIX mesh and P4HB plugs, are typically fully resorbed within 12–18 months [42]. Studies have shown that in a subcutaneous environment, the degradation rate of P4HB is slower than that of polyglycolic acid (PGA) but faster than that of poly(L-lactic acid) (PLLA) and poly(ε-caprolactone) (PCL). The degradation kinetics of P4HB are further governed by its physical structure. For instance, in a juvenile sheep model for pulmonary artery patch augmentation, the mass loss of implanted P4HB patches was positively correlated with surface porosity, suggesting that the in vivo degradation rate is influenced by the available surface area. The cleaving of hydrolytically unstable bonds in the polymer backbone results in a gradual erosion of the polymer surface and an overall decrease in molecular weight, which eventually break the mechanical integrity of the bulk material. Erosion is more like a beginning process of bioplastic hydrolysis, which is a physical process normally started from the bioplastic surface. Erosion is determined by parameters such as the attachment of biological materials (important), diffusion rates, and kinetics of the hydrolysis itself. The further degradation results in the formation of constituent monomers. Moreover, unlike synthetic absorbable polymers such as PGA, which can undergo a sudden and significant loss of mechanical strength, P4HB implants exhibit a more gradual change in its mechanical properties. This controlled degradation enables the release of 4-hydroxybutyrate (4HB) into the bloodstream, thereby preventing local acidification and the systemic accumulation of acidic byproducts. Consequently, the gradual biodegradation of P4HB into well-tolerated natural metabolites confers a superior in vivo stability profile, representing a significant advancement in the fabrication of medical implants and scaffolds [43,44].

It is notable that P3HB can be biodegraded in various environments, including aerobic conditions (e.g., soil) and anaerobic conditions (e.g., sludge), as well as in freshwater and saltwater. Examples of this include degradation in soil, marine degradation (ASTM D6691-17 for floating plastics [45], ASTM D7473-12 for buried plastics [46], and ASTM D7991-15 for tidal zone plastics [47]), and composting (ASTM D5338-15 [48]).

**Figure 4:**
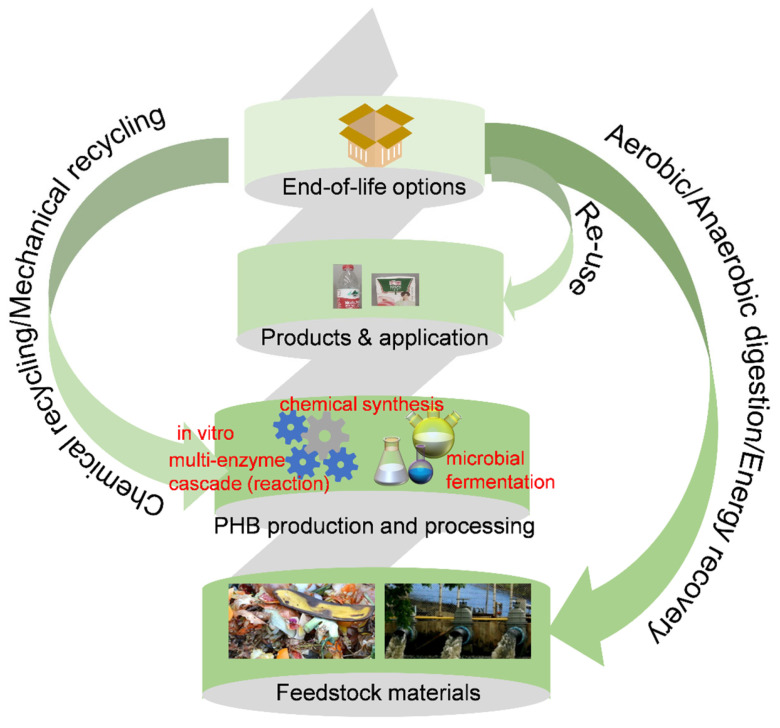
PHB life cycle assessment (LCA) studies.

Anaerobic degradation mainly encompasses landfill composting. The biodegradation of PHAs produces carbon dioxide and water as the main end products, along with small organic molecules. These products are generated during both aerobic and anaerobic degradation processes, depending on the conditions such as composting, landfill, and aquatic environments. Degradation tends to propagate rapidly in the amorphous PHB regions compared to the crystalline regions; therefore, it is crucial to investigate the degradation related to both amorphous and crystalline regions of the PHB, with the PHB LCA illustrated in Figure 4 [49].

### 3.1. Microbial and Enzymatic Degradation

The degradation mechanism of PHB can be divided into intracellular and extracellular types. Intracellular degradation is a cyclic process that varies with nutritional conditions. The key enzyme, 3-ketothiolase, bidirectionally regulates both synthesis and decomposition. Amorphous PHB granules are broken down by depolymerase into small molecules, which then enter the cellular metabolic pathway. Extracellular degradation primarily relies on enzymatic degradation mechanisms, where numerous bacteria, actinomycetes, and molds can secrete extracellular depolymerases to act on extracellular PHB. Microbial degradation is the mechanism that degrades PHB through the process of random hydrolysis of the material’s ester bonds in the polymer chain, leading to the complete degradation of the material and formation of CO_2_ and water [50]. This coincides with the “green” chemistry concept, and the degraded products can be recycled again. Enzymatic hydrolysis of PHB is a surface reaction, where the enzymes can attack the polymer free points [51]. The resulting water-soluble oligomers and monomers are accumulated in a medium. First, the enzymes attach to the PHB surface and then hydrolytically cleave the polymer, yielding only the non-toxic, water-soluble monomer R-3-hydroxybutyric acid, so the process involves safe, closed-loop recycling.

In recent years, significant progress has been made in understanding the mechanisms of PHB degradation, identifying key degrading microorganisms and characterizing the degradation behavior of PHB in different environments [52]. Based on their mode of action, PHB depolymerases can be divided into two types: endo- and exo-acting enzymes. Endo-acting depolymerases randomly hydrolyze ester bonds within the PHA polymer chain, producing oligomers and monomers, reducing the molecular weight of the polymer, and making it more susceptible to further degradation. Exo-acting depolymerases hydrolyze ester bonds from the ends of the PHA polymer chain, releasing monomers or dimers, and are key to the complete mineralization of PHB [53]. Professor Jendrossek and his team have conducted in-depth research on the structure and function of PHB depolymerases, elucidating the active sites, substrate specificity, and interaction mechanisms of these enzymes with the PHB surface [54].

The biodegradation behavior of PHB in various natural environments has been extensively studied. In soil environments, the degradation rate of PHAs is influenced by temperature, humidity, pH, and microbial communities. Due to high temperature and humidity, PHB degrades significantly faster in tropical regions compared to temperate regions. For example, the half-life of PHA films in tropical soils can be as short as a few weeks, whereas in temperate soils it may take several months [55]. In aquatic environments, the degradation rate of PHB in freshwater, seawater, and estuarine environments is affected by salinity, temperature, and dissolved oxygen. Generally, PHB degrades more slowly in marine environments than in soils, but degradation rates increase significantly under high-temperature conditions in the summer. In extreme environments, such as permafrost, acidic soils, mangroves, and coniferous forests, PHB also shows certain degradation capabilities, although the rates are typically slower.

### 3.2. Key Degrading Microorganisms and Their Mechanisms

A variety of microorganisms capable of degrading PHAs, including bacteria, fungi, and actinomycetes, have been isolated from different environments. Among them, bacteria are the most important degraders. For example, Pseudomonas lemoignei was the first PHA-degrading bacterium discovered and can secrete multiple types of PHB depolymerases [56]. Currently, about 360 microorganisms are known to synthesize PHA, ranging from 40 to 60 species of microorganisms according to various sources [57]. These microorganisms secrete extracellular depolymerases that hydrolyze PHB polymer chains into oligomers and monomers, which are further metabolized into carbon dioxide and water, achieving complete mineralization of PHAs. Based on extensive experimental data, researchers have developed predictive models for PHA degradation under various environmental conditions. These models can predict the degradation rate and half-life of PHB in specific ecosystems, providing a scientific basis for the design and application of PHB materials. Future research should focus on:

1. Molecular-level analysis of degradation mechanisms: in-depth study of the structure and function of PHA depolymerases to elucidate their interaction mechanisms with PHB surfaces.
2. Effects of environmental factors: systematic investigation of the influence of temperature, humidity, pH, salinity, and other environmental parameters on PHB degradation.
3. Regulation of microbial communities: exploring methods to enhance PHB degradation efficiency by modulating microbial community structures.
4. Optimization of predictive models: integrating big data and machine learning technologies to improve the accuracy and applicability of PHB degradation prediction models.

## 4. Current Industry Processing Approaches

In the industry, PHB is commonly processed using conventional methods such as injection molding [58], extrusion, and calendering [59,60], facilitating the production of a variety of products ranging from packaging materials to disposable items [61]. Particularly, the extrusion process is extensively utilized to prepare PHB for a variety of applications. The process involves melting the polymer and forcing it through a die to form filaments or sheets, which can then be further processed as needed [62]. Poly(hydroxybutyrate) (PHB) demonstrates notable resilience and adaptability during thermal processes. During thermal processing, PHB’s molecular weight could be reduced due to limited thermal stability. Moreover, the incorporation of polymers like poly-(3-hydroxyvalerate) and poly-(3-hydroxyhexanoate) enhances PHB’s thermal stability, expanding its processing window while also decreasing the molecular weight of these copolymers during thermal processing [63]. In the current industrial landscape, PHB processing is predominantly focused on enhancing the material’s physical properties and expanding its application range. Techniques like injection molding are employed to produce precise and intricate shapes, making PHB suitable for manufacturing components in the medical and agricultural sectors [64]. Additionally, film blowing is another prevalent method, especially for creating thin films used in packaging application [60]. Thus the wide range of PHB processing methods highlight the polymer’s flexibility in industrial applications [65,66].

### 4.1. Additive Manufacturing Processing

The advent of additive manufacturing (AM) has fundamentally transformed the production of various structures utilizing polyhydroxybutyrate (PHB) and its blends, markedly enriching the biocompatible materials pool accessible for a multitude of applications, particularly in biomedical and healthcare applications such as scaffolds, stents, suture, and mesh. Thus, with FDM technology, the polymer is subjected to multiple melting: at the first stage, granules are obtained from the flake-like polymer isolated from microbial cells (first melting); then, the filament (rod-like strands) required for 3D printing is obtained from the granules by melt extrusion (second melting); and finally, the product is printed from the filament via layer-by-layer fusion deposition technologies (third melting) [26]. Table 2 summarizes the additive manufacturing processing of PHB-based materials, structures, and their applications. The table lists various PHB composites and blends, such as PLA/PHB-organoclay composite, PHB-BaTiO_3_ nanocomposite, and PHBV-ZrO_2_ composite, along with the processing methods used, such as fused deposition modeling (FDM), direct powder extrusion (DPE), and stereolithography (SLA). It also details the structures produced, including porous scaffolds, films, and conductive traces, and their applications in fields like tissue engineering, regenerative medicine, and pharmaceuticals. This table provides a comprehensive overview of the versatility of PHB materials in AM, highlighting the relationship between material composition, processing method, and end-use application.

#### 4.1.1. Fused Deposition Modeling (FDM)

PHB polymers are typically transformed into filament via extrusion, which is then utilized in fused deposition modeling (FDM), one of the most adopted thermoplastic 3D printing techniques. The filament preparation process involves heating the polymer and pushing it through a nozzle to produce continuous feedstock for printing. This extrusion process determines the material’s consistency and quality, which are essential for successful 3D printing. However, the thermal processing of PHB polymers through FDM presents challenges. PHB alone has a narrow processing window, leading to issues such as filament breakage and difficulties in controlling filament size during melt extrusion. Consequently, PHB is often composited with fillers or blended with other organic substances to improve its printability [26].

Selected fillers can improve the overall processibilities and performances of the 3D-printed structures. Blending PHB with organic substances, including polymers and small molecule plasticizers, was found to be an effective way to improve processibilities. Kanabenja et al. showed that the addition of polypropylene glycol (PPG) as a plasticizer in the PHB/PLA blend, reinforced with hydroxyapatite (HA), significantly enhances the processability and mechanical properties of bio-composite filaments, making them a viable alternative to commercial PLA for 3D printing applications [69]. Sahin et al. also reported that blending PLA and PHB helps improve the quality and tensile strength of 3D-printed specimens [72]. D’Arienzo et al. reported the use of cellulose fibers as micro-filler that significantly improves the thermal stability and mechanical performance of the resulting PHB-cellulose composites since the cellulose microfibers strengthen the ester bond and then form a strong network [71]. Li et al. investigated the use of multiwalled carbon nanotubes (MWCNTs) as additives and discovered that they enhance the material properties of PHB, including conductivity and biocompatibility [73]. Furthermore, they demonstrated that 3D-printed PHB/MWCNTs composites can form porous structures that are suitable for tissue engineering applications.

#### 4.1.2. Powder/Gel Extrusion

A recent alternative to FDM is direct powder extrusion (DPE). DPE processes physical blends of materials in the form of pellets or powders through a hot melt extruder in the printer. This method eliminates the intermediate filament production steps and requires only a small amount of materials. Moroni et al. reported DPE 3D printing of PHB blended with acetaminophen, a model drug, for prolonged release and personalized drug delivery forms [76]. Gel formation was achieved either through the extraction of PHA from dried biomass or via dissolution in organic solvent, with subsequent cooling to ambient temperature [77]. The resulting gels were pressed to remove excess solvent, yielding compact gel cakes, or can be patterned using direct ink printing. Notably, the gels were characterized by a significant reduction in PHA melt temperature [78,79].

#### 4.1.3. Stereolithography (SLA)

The SLA process is a 3D printing technique that uses resin photopolymerization. In this method, a laser activates photo-initiators in a resin comprising monomers and oligomers, triggering a cross-linking reaction that solidifies the structures. SLA has been adapted for manufacturing PHB-based blends; although PHB lacks photo-active groups, it can be processed by blending with photoactive resins. Bakar et al. reported on using SLA to manufacture casts for bone fractures by incorporating urethane dimethacrylate (UDMA) with photo-initiators and PHB, creating a printable resin with suitable viscosity [2]. However, adding 13% PHB increases the viscosity beyond the threshold suitable for flow-based printing.

#### 4.1.4. Electrospinning

Electrospinning of Polyhydroxybutyrate (PHB) is an interesting area of research with potential in various applications, especially in biomedical areas and environmental sustainability. The process involves the application of a high voltage to create an electrically charged jet of polymer solution or melt, which is then stretched and elongated into fine fibers. This method allows for the production of fibers with diameters ranging from a few nanometers to several micrometers. The process involves dissolving PHB in a suitable solvent (e.g., chloroform, dichloromethane) to form a spinning solution. The viscosity and concentration of the solution, as well as processing parameters such as voltage, flow rate, and distance from the needle to the collector, influence the morphology and diameter of the fibers produced [80]. Electrospun PHB nanofibers have shown promise in wound dressing materials, scaffolds for tissue regeneration, and drug delivery systems due to their biocompatibility and ability to mimic the extracellular matrix. PHB-based nanofibers can be used in environmental applications such as water filtration and soil erosion control due to their biodegradability and sustainable sourcing [81,82]. Despite its potential, electrospinning of PHB faces challenges such as controlling fiber morphology and achieving consistent quality at large scale. Issues related to solvent toxicity and the need for biocompatible solvents also need to be addressed for biomedical applications. Future research directions include enhancing the mechanical properties of electrospun PHB, exploring new applications in areas such as energy storage devices and flexible electronics, and optimizing the electrospinning process for industrial scalability [83].

## 5. PHB Composites

### 5.1. Blends Using Biopolymers

Poly (lactic acid) or polylactide, a bio-source based polyester, is made from lactic acid through polymerization using first generation feedstock (e.g., corn, wheat, and sugarcane). PLA has been largely used in additive manufacturing with good flexibility, toughness, and copolymers with other plastics, rubber, nanofillers, etc., for advanced functions [26]. It has also been certified as a safe biopolymer by the United States Food and Drug Administration (FDA) [75]. Main PLA manufacturers are NatureWorks LLC, Pyramid Bioplastics, Cargill, and Galactic. Natural Works Inc. produces powders, pellets, and filaments available on the market. PLA is an inexpensive biopolymer compared to other biopolymers such as PHB, and commercially available PLA products include straws, bags, and containers found in daily life. While PLA is still more costly than PP and PS, PLA degrades slowly (elevated temperature needed) and produces CO_2_ during the degradation process. This issue offsets the fact that PLA is derived from renewable resources and that some of the gases are absorbed and fixed; therefore, detailed LCA studies on PHB/PLA blends are needed.

#### 5.1.1. PCL and PBAT

PCL is derived from synthetic feedstock but is biodegradable and has been utilized intensively in shape memory biomaterials owing to its low Tg and great processibility. PHB/PCL blends have been incorporated with plasticizers, such as oils and silica particles, to increase mechanical strength and elasticity. Some drugs, for example, antibiotics, have been incorporated with PHB/PCL composites for drug delivery use. A future research direction for PHB/PCL blends would be to make artificial tissue textures for biomedical uses, such as regenerating bone, tendon, and cartilage [84]. Polybutylene Adipate Terephthalate (PBAT) is a fully biodegradable, synthetic thermoplastic polyester. It is commercially prominent as a flexible and compostable polymer, primarily designed to replace conventional, non-biodegradable plastics like polyethylene in applications such as packaging and agricultural films. PBAT is classified as a bioplastic; however, it is important to note that it is typically derived from fossil-based precursors, not renewable biomass. PBAT and PHB composites have been studied to improve their biodegradability and enhance their brittleness using the 3D printing method [85]. A PHB and PBAT blend has been fabricated using the melt-blowing method to address low efficiency and high consumption of PHB. This design increased the specific surface area and porosity of the blown films, with an average pore diameter of 59.66 μm and a fiber diameter of 11.61 μm to be used for solid-phase denitrification [86].

PHBV was blended with oil and dissolved in solvents for coatings on top of tissue papers. The resulting coated paper can be utilized as a green-based food packaging material (e.g., coffee container) instead of wax coating [87]. Martinez-Sanz et al. used solvent casting to prepare PHBV/BCNW films, showing four times more oxygen and water vapor barrier properties than neat PHBV with potential use in plastic wraps [88] Abdalkarim et al. reported that 10% CNC-ZnO/PHBV composites exhibited improvements to mechanical, barrier, and antibacterial properties [89].

#### 5.1.2. Nanoclay-Based

The clay/PHBV nanocomposites modified through solvent processing could be used in packaging with enhanced water-barrier properties. Wu et al. systematic study was conducted on blending PHBV with PBAT and nano-clay toughening and reinforcing agents to address its inherent brittleness and poor gas barrier performance. The results demonstrated a significant improvement in the toughness, ductility, and gas barrier properties of PHBV [90].

#### 5.1.3. Natural Fibers

PHBV composites reinforced with natural fibers have been investigated for the potential use in respiring food products and water sensitive products, and the manufacturing approaches are mainly through injection molding and extrusion process for large scale production. The resulting materials exhibited degraded mechanical properties, while the purpose of adding natural fibers is mainly for lowering the expense while maintaining the PHBV properties as much as possible. Angellier et al. investigated the wheat straw size, morphology, and content of PHBV composites, focusing on the resulting mechanical properties and water and oxygen permeability; the findings suggest that small fiber size and less fiber mass fraction in the PHBV matrix content is preferred when preparing flexible composites [91,92]. Cellulose crystals/PHBV presented enhanced barrier properties against oxygen and water vapor for potential use in packaging applications [93]. CNC, which stands for cellulose nanocrystals, are nanoscale crystalline particles derived from cellulose. They are known for their high strength, stiffness, and biodegradability. In the context of PHBV, CNC has been used as a reinforcing agent to improve the mechanical properties of PHBV-based composites. For instance, studies have shown that incorporating CNC into PHBV can enhance its tensile strength and modulus, making the composite material more suitable for various applications.

#### 5.1.4. Natural Rubber

Natural rubbers have also been used in melt blending with PHBV to enhance the toughness and flexibility of the plastic matrix, with a reported tensile strength of 25 MPa and 8% increase in elongation at break. The products showed comparable sealability and water vapor permeability to polypropylene and were reported to be food-contact safe; however, the composite colors were dark brown, which have to be adjusted in further investigations [94].

### 5.2. Plasticizers

One of the issues in using PHB is its brittleness and, therefore, many studies have been carried out with the inclusion of bio-plasticizers in PHB matrices in hopes of overcoming this drawback. Examples of bio-plasticizers modified with PHB include glycerol, oxypropylated glycerin, polyethylene glycol (PEG), acetyl tributyl citrate (ATBC), soybean oil, and epoxidized soybean oil. These compounds were selected based on their high availability, low cost, and natural origin [95,96].

Plasticizers will continue to display an important part in our economy, ecosystems, and marine environment. Plasticizers are often low-molecular-weight substances blended to polymers to facilitate processability, toughness, and flexibility by lubricating the polymer chains or being obstacles in forming crystalline regions in polymers [97]. Adding plasticizer to the PHB matrix may be an effective and simple approach to reduce its brittleness by interacting with PHB’s amorphous regions, reducing secondary bonds interacting with polymer chains, and in turn lowering the overall crystallinity within the polymer. Plasticizers may act as lubricants inside polymer chains to increase chain mobility, resulting in decreases in Tg and Tm of the polymer, providing an improved processing window for the materials, and prolonging the thermal processing window of PHB. Overall, the incorporation of plasticizing materials into polymers is a relatively viable approach that helps reduce costs and improve processability. Sometimes the inclusion of additives brings new problems in resulted products, for instance, plasticizers in PHB have been found to cause decreases in tensile properties and the modification of degradability; therefore, these factors have to be well investigated and controlled based on the expected properties of final polymeric products. Glycerol acts as a plasticizer to lubricate the polymer chains through an alcoholysis reaction; at 20 wt%, it is optimal for toughening PHB since fragmented chains recombine and result in enhanced mechanical ductility [98,99]. The Table 3 shows the PHB blends and the resulted mechanical properties.

Exothermic crystallization is a key process in the solidification of PHAs. During crystallization, heat is released, and understanding the kinetics of this exothermic process is crucial for controlling the cooling rate during processing. A faster cooling rate can lead to the formation of smaller spherulites, which are spherical crystalline structures in the polymer matrix. Smaller spherulites generally result in improved mechanical properties, such as higher tensile strength and toughness, as they can better distribute stress within the material.

The rates of formation and morphology of spherulites are influenced by several factors, including the type of PHA, the presence of additives, and the processing conditions [115]. For example, the addition of nucleating agents can significantly increase the rate of spherulite formation and reduce their size. Different processing methods, such as injection molding and extrusion, can also affect spherulite morphology due to variations in shear stress and cooling rates. Optimizing these characteristics is essential for improving the durability of PHA-based products. By controlling the crystallization process, it is possible to reduce the risks of repeated crystallization processes, which can lead to dimensional changes and mechanical property degradation over time. Additionally, understanding the relationship between crystallization and biodegradation is important for regulating the rates of biodegradation. For instance, a more crystalline PHA may degrade more slowly than an amorphous one, and by adjusting the crystallization behavior through blending and processing, it is possible to design materials with specific biodegradation rates for different applications [38]. In conclusion, a comprehensive approach that considers the use of copolymers, blends with various materials, and a detailed understanding of thermal and crystallization characteristics is necessary for fully exploiting the potential of PHAs.

## 6. Applications

**Figure 5:**
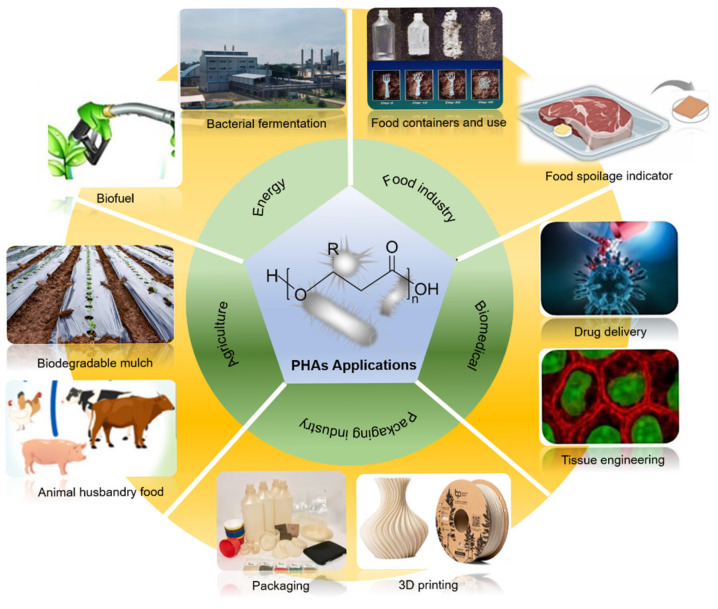
PHB applications [118,119,120,121].

Biopolymers are utilized in multiple areas such as eco-friendly packaging, advanced biological implants, biological and medical applications, and disposable electronics and sensors, since biopolymers naturally respond to biological matter, exhibited in Figure 5 [116,117].

### 6.1. Traditional Use-Food Packaging

Food packaging is a primary sector for the end use of plastics; to maintain the hygiene and integrity of packaged food during use, transportation, and degradation, several factors have to be considered when choosing packaging materials. These properties mainly refer to chemical, physical, thermal, mechanical, optical, and oxygen/moisture characteristics. PHA for bioplastic packaging is one environmentally benign option in line with the concept of carbon reduction.

The thermal properties of Tg and Tm in plastics are important in selecting food packaging materials, where Tg should be low enough as not to fail in freezer storage and Tm should be high enough to withstand maximum temperature use. Plastics with a low Tg are beneficial in food packaging for freezing storage, but their uses in hot food packaging are limited. PHB has a relatively low Tg and often perform poorly in terms of thermal stability under high temperatures (>120 °C). Improving the heat resistance of PHAs (e.g., PHB/wax composites) effectively expands their use and facilitates replacement of conventional plastics.

PHB has tensile properties similar to conventional plastics, although PHAs’ elongation at break (normally < 5%) is inferior to conventional plastics. A ductile food packaging is often desired, and this property can be achieved by adding plasticizers and designing composites. The barrier properties for food packaging applications refer to the resistance to oxygen and moisture to maintain food freshness and prolong shelf life. PHAs are permeable to small molecules such as oxygen, carbon dioxide, water vapor, organic vapors, and some liquids, with typical barrier properties to oxygen and water moisture. PHB had a good oxygen barrier property (2–10 cm^3^ mm m^−2^ 24 h^−1^ atm^−1^) compared with other bioplastics, and thus, increasing the processability of PHAs would promote sustainable packaging industries [122].

### 6.2. Emerging Applications

One of the emerging areas for PHB is the biomedical field since it has been FDA approved bio-safe for medical use and has been utilized as TephaFLEX absorbable sutures [123]. Other promising applications include stents, surgical meshes, scaffolds, heart valves, wound dressing, medical adhesives, and tapes. Some PHB materials suffer from high manufacturing cost and poor processability.

Recently, researchers have been intensively investigating PHA scaffolds and membranes used for bone tissue engineering through diverse manufacturing processes [124]. Electrospinning, solvent casting, and additive manufacturing, including material extrusion and traditional fused deposition modeling, have been used for fabricating PHB membranes, films, and scaffolds (discussed in the Section on Electrospinning). P4HB-based scaffolds are particularly well suited for soft tissue applications, as they provide mechanical support while concurrently enabling robust tissue ingrowth. Furthermore, the material is resorbed by the body in a predictable and steady manner. Other emerging PHB applications have been found in additives for textiles and in food for fish and prawn, without causing aquatic toxicity [125].

With the growing awareness of environmental protection, the applications of PHB in multiple fields are becoming increasingly widespread and drawing significant attention. In the realm of eco-friendly materials and composites, PHB can be compounded with natural fibers such as hemp fiber, bamboo fiber, and wood powder to produce environmentally friendly wood-plastic composites. These composites not only possess good mechanical properties but also achieve complete biodegradability, finding extensive applications in outdoor flooring, landscape gardening, and architectural decoration [126]. In the agricultural sector, remarkable progress has been made in the applications of PHB. PHB agricultural mulch films can naturally degrade after use, effectively avoiding the “white pollution” problem caused by traditional plastic mulch films. Additionally, PHA can be used to manufacture seedling containers and fertilizer coating materials, providing strong support for sustainable agricultural development [127]. In the field of textiles and fibers, PHA fibers exhibit excellent breathability and comfort. They can be used alone or blended with natural fibers such as cotton and hemp to produce biodegradable textiles, including eco-friendly clothing and medical textiles. As technology advances, the performance of PHB fibers continues to improve, offering a promising future in the high-end textile industry [128]. The applications of PHB in the automotive and home furnishing sectors are also gradually emerging. PHB can be utilized to manufacture automotive interior components, sound insulation materials, and household items. These products not only demonstrate good performance during use but also enable environmentally friendly disposal at the end of their service life, aligning with the requirements of modern society for sustainable development.

## 7. Discussion

PHAs are of great importance biopolymers derived from biological sources and can be completely degraded in a relatively short time. The main barrier to the wide use of PHAs is its inferior mechanical properties compared to currently used plastics, such as PE and PP. In addition, the high cost of the microbiological route for large-scale PHA production has impeded the use of PHAs. Cost reduction can be achieved through the isolation of robust native microbial producers and the genetic engineering of strains for efficient synthesis, building upon the significant scientific progress reviewed herein. A major challenge remains in the high-yield production of P4HB from inexpensive, structurally unrelated carbon sources, a feat currently only attainable in recombinant strains. Furthermore, advances in bioprocess engineering and the development of environmentally friendly, scalable extraction methods that yield polymers of various purities (including endotoxin-free grades) are essential to enhancing productivity and lowering costs. Expanding the utility of P4HB also depends on tailoring its molecular weight for specific applications. While ultra-high-molecular-weight P4HB can be processed into high-strength fibers, low-molecular-weight variants are suitable for drug delivery or as plasticizers.

Therefore, the aim should focus on lowering production costs of fabricating inexpensive PHA blends, on finding strong bacterial strains, low-cost carbon sources from an upstream production standpoint, processing techniques, cost-effective blends, and strong bacterial strains for biodegradation. Advanced genetic engineering and synthetic biology approaches are needed for developing new bacterial strains and low-cost carbon sources for waste. Also, advanced techniques for efficient PHA extraction and purification are important to reduce PHA production cost. Moving forward, efforts will focus on optimizing PHA production processes to enhance cost-effectiveness and scalability, thereby fostering their integration into a circular economy where materials are reused, recycled, or composted at the end of their lifecycle.

Future research will explore novel composite formulations and manufacturing techniques to achieve optimal performance while maintaining sustainability. Despite their promise, PHAs face several challenges that must be addressed to realize their full potential:

1. Cost Competitiveness: PHA production costs are currently higher than those of traditional plastics. Research efforts will focus on improving fermentation processes, exploring alternative feedstocks, and scaling up production to reduce costs and increase competitiveness.
2. Performance Optimization: Enhancing the mechanical, thermal, and barrier properties of PHA-based materials through advanced processing techniques and innovative additives remains a critical area of research.
3. Regulatory Frameworks: Developing supportive regulatory frameworks and standards for PHAs, particularly regarding biodegradability claims, actual on-site GWP values data, and end-of-life management, will be essential to ensure market acceptance and environmental impact assessment consistency.

By harnessing the inherent properties of PHAs and leveraging composite technologies, we can create a more sustainable and resilient future, where materials are not just functional but also environmentally responsible. As stakeholders across academia, industry, and policy-making collaborate, PHAs are poised to play a pivotal role in shaping a circular economy and reducing our dependence on fossil-based plastics.

## Figures and Tables

**Table 1:** General properties for P(3HB) and P(4HB).

	P(3HB)	P(4HB)
Melting Temperature, Tm (°C)	175	60
Glass Transition Temperature, Tg (°C)	4–10	−51
Density, p (g/cm^3^)	1.18–1.26	1.17–1.22
Crystallinity (Xc, %)	65–80	20–35
Young’s Modulus, E (GPa)	1.4–3.5	0.07
Ultimate Tensile Strength (MPa)	15–40	50–70
Elongation at Break (%)	4–10	1000

**Table 2:** Additive manufacturing of PHB-based structures.

Material	Processing Method	Structure Produced	Application	References
PLA/PHB-Organoclay composite	FDM	-	-	[67]
PHB-BaTiO_3_ Nanocomposite	FDM	porous cubic scaffold	vascularized bone tissue engineering	[68]
PHB/PLA-hydroxyapatite composite	FDM	-	-	[26,69]
PHBV-ZrO_2_ composite	FDM	porous scaffold	regenerative medicine	[70]
PHB–cellulose composite	FDM	-	-	[71]
PHB-graphite composite, PHB/PLA blend	FDM	dog-bone specimen	-	[72]
PHB-MWCNTs composite	FDM	scaffold, conductive traces	Tissue regeneration	[73]
PLA/PHB blend	FDM	scaffold	Medical applications	[74]
PHB/PUA blend	FDM	finger splint cast	medical devices	[74]
PHB/PLA-Kaolin composite	FDM	-	-	[75]
PHB/acetaminophen	DPE	cubic structure	pharmaceutical forms	[76]
Urethane Dimethacrylate/PHB	SLA	fracture bone cast	temporary medical devices	[2]

**Table 3:** Blends and mechanical performance.

Blends	Youngs’ Modulus (MPa)	Tensile Strength (MPa)	Elongation at Break (%)	Use	Source
PLA/PHBV (80/20)/0.3 wt% 2,5-dimethyl-2,5-di(tert-butylperoxy)hexane (DBPH)		-	15.95	-	[100]
PE/PHBV (80/20, 70/30)	348.39 + 12.5 299.7 + 8.67	25.93 + 0.72 17.5 + 0.3	-	Packaging, oxygen transmission rate (~1200 cm^3^/cm^2^ per day)	[101,102]
PHBV/PBS (50/50)	1900	36	-	-	[103]
PHB/PEG (9:1)	430.97 ± 31.09	12.57 ± 1.09	3.34 ± 0.93	antimicrobial packaging	[104]
PHB/PEG (8/1)	254.4 ± 26.7	3.4 ± 0.3	24 ± 6	air filtration or water filtration	[104]
PHB-5% TABC	-	14.8	6.3		[105]
PHBV-5%MFC/epoxidized soybean oil	2670.2 ± 21.7	27.3 ± 1.2	1.27 ± 0.11	packaging	[106]
starch/12%PHA	-	3.75	72.4	packaging	[107]
(maleic anhydride-grafted polyhydroxyalkanoate) PHA-g-MA/(palm fiber) TPF	338	12.9	-	imitation wood, and in medical and conductive filaments	[108]
PHA-g-MA/TPF	424	23.7	-	-	[109]
PHA/50% (polycaprolactone) PCL	280	6.4	51.9	plastic	[110]
boric acid cross-linked starch/PHA (cross-linking agent: boric acid)	-	8.55	38.6	packaging	[111]
PHA/(siliceous sponge spicules) 2% SSS	342	15	518	biomedical material	[112]
PHA-g-(Acrylic acid) AA/2% SSS	372	22	565	-	[112]
1% (Cellulose nanocrystals) CNC/PHA	720 ± 20	22.5 ± 0.02	10.43 ± 0.23	paper	[113]
PHA/20% (cellulose microfibers) MF	940 ± 0.14	24.9 ± 0.05	3.78 ± 0.11%	paper	[114]

## Data Availability

The original contributions presented in this study are included in the article. Further inquiries can be directed to the corresponding authors.

## References

[1] Zhao X., Wang Y., Chen X., Yu X., Li W., Zhang S., Meng X., Zhao Z.M., Dong T., Anderson A. (2023). Sustainable bioplastics derived from renewable natural resources for food packaging. Matter.

[2] Abu Bakar A.A., Zainuddin M.Z., Abdullah S.M., Tamchek N., Mohd Noor I.S., Alauddin M.S., Alforidi A., Ghazali M.I.M. (2022). The 3D Printability and Mechanical Properties of Polyhydroxybutyrate (PHB) as Additives in Urethane Dimethacrylate (UDMA) Blends Polymer for Medical Application. Polymers.

[3] Morris M.R., Stanton A., Blomberg T., Hicks A. (2024). Human behavior outcomes at point of disposal of a biodegradable plastic cup at a U.S.-based university campus. Resour. Conserv. Recycl..

[4] Benavides P.T., Dunn J.B., Han J., Biddy M., Markham J. (2018). Exploring Comparative Energy and Environmental Benefits of Virgin, Recycled, and Bio-Derived PET Bottles. ACS Sustain. Chem. Eng..

[5] Müller Carneiro J., Figueirêdo M.C.B., Rodrigues C., Azeredo H.M.C., Freire F. (2023). Ex-ante life cycle assessment framework and application to a nano-reinforced biopolymer film based on mango kernel. Resour. Conserv. Recycl..

[6] Lee J.G., Raj R.R., Day N.B., Shields C.W.I. (2023). Microrobots for Biomedicine: Unsolved Challenges and Opportunities for Translation. ACS Nano.

[7] Smith R.L., Takkellapati S., Riegerix R.C. (2022). Recycling of Plastics in the United States: Plastic Material Flows and Polyethylene Terephthalate (PET) Recycling Processes. ACS Sustain. Chem. Eng..

[8] Dietrich K., Dumont M.J., Del Rio L.F., Orsat V. (2017). Producing PHAs in the bioeconomy-Towards a sustainable bioplastic. Sustain. Prod. Consum..

[9] Fu X., Xu H., Zhang Q., Xi J., Zhang H., Zheng M., Xi B., Hou L. (2023). A review on polyhydroxyalkanoates production from various organic waste streams: Feedstocks, strains, and production strategy, Resources. Conserv. Recycl..

[10] Chandra R., Rustgi R. (1998). Biodegradable polymers. Prog. Polym. Sci..

[11] Manavitehrani I., Fathi A., Badr H., Badr H., Daly S., Shirazi A.N., Dehghani F. (2016). Biomedical applications of biodegradable polyesters. Polymers.

[12] Ojumu T.V., Yu J., Solomon B.O. (2004). Production of polyhydroxyalkanoates, a bacterial biodegradable polymers. Afr. J. Biotechnol..

[13] Utsunomia C., Ren Q., Zinn M. (2020). Poly(4-Hydroxybutyrate): Current State and Perspectives. Front. Bioeng. Biotechnol..

[14] Macedo M.A.D., Oliveira-Filho E.R., Piccoli R.A.M., Gomez J.G.C., Silva L.F. (2024). Poly(3-hydroxybutyrate-co-4-hydroxybutyrate) [P(3HB-co-4HB)] biotechnological production: Challenges and opportunities. Biomass Convers. Biorefin..

[15] Mitra R., Xiang H., Han J. (2021). Current Advances towards 4-Hydroxybutyrate Containing Polyhydroxyalkanoates Production for Biomedical Applications. Molecules.

[16] Hou X.A., Sun Wen Liu Z.B., Liu S.Q., Chee J., Yeo C., Lu X.H., He C.B. (2022). Tailoring crystalline morphology via entropy-driven miscibility: Toward ultratough, biodegradable, and durable polyhydroxybutyrate. Macromolecules.

[17] Ding Y., Li M., Dong W., Kan Z., Li Z.B. (2025). Favorable compatibility efficiency and thermal stability of PLA/P4HB/PGMA blends contributed by phase interface-located chain expansion reaction. Polym. Degrad. Stab..

[18] Visco A., Scolaro C., Facchin M., Brahimi S., Belhamdi H., Gatto V., Beghetto V. (2022). Agri-food wastes for bioplastics: European prospective on possible applications in their second life for a circular economy. Polymers.

[19] Zhang Z., Gowda R.R., Chen Y.X. (2024). Chemosynthetic P4HB: A Ten-Year Journey from a Non-Polymerizable Monomer to a High-Performance Biomaterial. Acc. Mater. Res..

[20] Lemoigne M. (1927). Produits de déshydratation et de polymérisation de l’acide β-oxobutyrique. Bull. Société Chim. Biol..

[21] Kumar M., Rathour R., Singh R., Sun Y.Q., Pandey A., Gnansounou E., Lin K.Y.A., Daniel C.W., Tsang Thakur I.S. (2020). Bacterial polyhydroxyalkanoates: Opportunities, challenges, and prospects—ScienceDirect. J. Clean. Prod..

[22] Roblero M.A. (2021). Evaluation of Fed-Batch Fermentation for Production of Polyhydroxybutyrate with a Banana Pulp Juice Substrate from an Agro Industrial By-Product. Front. Sustain. Food Syst..

[23] Wang J.F., Huang J.Q., Liu S.J. (2024). The production, recovery, and valorization of polyhydroxybutyrate (PHB) based on circular bioeconomy. Biotechnol. Adv..

[24] Zytner P., Kumar D., Elsayed A., Mohanty A., Ramaraoc B.V., Misra M. (2023). A review on polyhydroxyalkanoate (PHA) production through the use of lignocellulosic biomass. RSC Sustain..

[25] Nath D., Misra M., Al-Daoud F., Mohanty A.K. (2025). Studies on poly (butylene succinate) and poly (butylene succinate-co-adipate)-based biodegradable plastics for sustainable flexible packaging and agricultural applications: A comprehensive review. RSC Sustain..

[26] Kanabenja W., Passornraprasit N., Aumnate C., Tim A., Osswald T.A., Aht-Ong D., Potiyaraj P. (2024). Enhancing 3D printability of polyhydroxybutyrate (PHB) and poly (3-hydroxybutyrate-co-3-hydroxy valerate) (PHBV) based blends through melt extrusion based-3D printing. Addit. Manuf..

[27] Modi S., Koelling K., Vodovotz Y. (2011). Assessment of PHB with varying hydroxyvalerate content for potential packaging applications. Eur. Polym. J..

[28] Zhao H., Cui Z., Sun X., Turng L.S., Peng X.f. (2013). Morphology and properties of injection molded solid and microcellular polylactic acid/polyhydroxybutyrate-valerate (PLA/PHBV) blends. Ind. Eng. Chem. Res..

[29] Choi S.Y., Cho I.J., Lee Y., Kim Y.J., Kim K.J., Lee S.Y. (2020). Microbial polyhydroxyalkanoates and nonnatural polyesters. Adv. Mater..

[30] Martin D.P., Williams S.F. (2003). Medical applications of poly-4-hydroxybutyrate: A strong flexible absorbable biomaterial. Biochem. Eng. J..

[31] Yang X.Y., Luo H.Q., Zhou R.Y., Wei C.Y., Deng J., Luo J.H., Yan X.F., Yu K., Yuan S., Zhou W. (2024). Miscibility, crystallization and morphology in the novel polylactide/poly(4-hydroxybutyrate) blends. Addit. Manuf..

[32] Williams S.F., Rizk S., Martin D.P. (2013). Poly-4-hydroxybutyrate (P4HB): A new generation of resorbable medical devices for tissue repair and regeneration. Biomed. Technol..

[33] Williams S.F., Martin D.P., Moses A.C. (2016). The history of GalaFLEX P4HB scaffold. Aesthetic Surg. J..

[34] Zhang M., Thomas N.L. (2011). Blending polylactic acid with polyhydroxybutyrate: The effect on thermal, mechanical, and biodegradation properties. Adv. Polym. Technol..

[35] Zelzer M., Ulijn R.V., Aguilar M.R., Román J.S. (2014). 6—Enzyme-responsive polymers: Properties, synthesis and applications. Smart Polymers and Their Applications.

[36] Vecitis C.D., Park H., Cheng J., Mader B.T., Hoffmann M.R. (2008). Kinetics and mechanism of the sonolytic conversion of the aqueous perfluorinated surfactants, perfluorooctanoate (PFOA), and perfluorooctane sulfonate (PFOS) into inorganic products. J. Phys. Chem. A.

[37] Dalton B., Bhagabati P., De Micco J., Padamati R.B., Connor K. (2022). A review on biological synthesis of the biodegradable polymers polyhydroxyalkanoates and the development of multiple applications. Catalysts.

[38] Koller M., Heeney D., Mukherjee A. (2025). Biodegradability of polyhydroxyalkanoate (PHA) biopolyesters in nature: A review. Biodegradation.

[39] Kalia V.C., Patel S.K.S., Lee J.K. (2023). Exploiting Polyhydroxyalkanoates for Biomedical Applications. Polymers.

[40] Keridou I., Franco L., Valle L.J.D., Juan C., Funk L., Turon P., Puiggalí J. (2021). Hydrolytic and enzymatic degradation of biobased poly(4-hydroxybutyrate) films. Selective etching of spherulites. Polym. Degrad. Stab..

[41] Fernandes Cavaleiro M.Â. (2023). Biodegradation of PHA/PBAT Packaging Materials by Soil Microorganisms. Ph.D. Thesis.

[42] Tran D.H., Rubarth C., Leeds S.G., Fair L., Gowan T.M., Ramakrishnan S., Shabbir R., Ogola G., Ward M.A., Aladegbami B. (2024). The use of poly-4-hydroxybutyrate (P4HB, Phasix™) mesh in ventral hernia repair: A systematic review and meta-analysis. Hernia.

[43] Koller M., Maršálek L., de Sousa Dias M.M., Braunegg G. (2016). Producing microbial polyhydroxyalkanoate (PHA) biopolyesters in a sustainable manner. New Biotechnol..

[44] Lenz R.W., Marchessault R.H. (2005). Bacterial Polyesters:  Biosynthesis, Biodegradable Plastics and Biotechnology | Biomacromolecules. Biomacromolecules.

[45] (2017). Standard Test Method for Determining Aerobic Biodegradation of Plastic Materials in the Marine Environment by a Defined Microbial Consortium or Natural Sea Water Inoculum.

[46] (2021). Standard Test Method for Weight Attrition of Non-Floating Plastic Materials by Open System Aquarium Incubations.

[47] (2015). Standard Test Method for Determining Aerobic Biodegradation of Plastics Buried in Sandy Marine Sediment under Controlled Laboratory Conditions.

[48] (2015). Standard Test Method for Determining Aerobic Biodegradation of Plastic Materials under Controlled Composting Conditions, Incorporating Thermophilic Temperatures.

[49] Dintcheva N. (2024). Overview of polymers and biopolymers degradation and stabilization towards sustainability and materials circularity. Polymer.

[50] Uefuji M., Kasuya K.-I., Doi Y. (1997). Enzymatic degradation of poly[(R)-3-hydroxybutyrate]: Secretion and properties of PHB depolymerase from *Pseudomonas stutzeri*. Polym. Degrad. Stab..

[51] Yoshie N., Oike Y., Kasuya K.-I., Doi Y., Inoue Y. (2002). Change of Surface Structure of Poly(3-hydroxybutyrate) Film upon Enzymatic Hydrolysis by PHB Depolymerase. Biomacromolecules.

[52] Akhlaq S., Singh D., Mittal N., Srivastava G., Siddiqui S., Faridi S.A., Siddiqui M.H. (2023). Polyhydroxybutyrate biosynthesis from different waste materials, degradation, and analytic methods: A short review. Polym. Bull..

[53] Kwon S. (2025). A Review of Biodegradation Mechanisms and Evaluation Methods for Biobased Materials: Polysaccharides, Lignin, and Biopolyesters. J. Korea TAPPI.

[54] Zhang X., Fevre M., Jones O.J., Waymouth R.M. (2018). Catalysis as an enabling science for sustainable polymers. Chem. Rev..

[55] Lott C., Eich A., Makarow D., Unger B., Eekert M.V., Schuman E., Reinach M.S., Lasut M.T., Weber M. (2021). Half-life of biodegradable plastics in the marine environment depends on material, habitat, and climate zone. Front. Mar. Sci..

[56] Jendrossek D., Frisse A., Behrends A., Kratzin H.D., Stanislawski T., Schlegel H.G. (1995). Biochemical and molecular characterization of the Pseudomonas lemoignei polyhydroxyalkanoate depolymerase system. J. Bacteriol..

[57] Zhang L., Tsui T.H., Fu J., Dai Y.J., Tong Y.W. (2022). Valorization of poly-β-hydroxybutyrate (PHB)-based bioplastic waste in anaerobic digesters of food waste for bioenergy generation: Reactor performance, microbial community analysis, and bioplastic biodegradation. Carbon Neutrality.

[58] Hernandez M.M., Gupta N.S., Lee K.S., Pital A.C., Marrone B.L., Iverson C.N., Dumont J.H. (2021). Characterization of Polyhydroxybutyrate-Based Composites Prepared by Injection Molding. Polymers.

[59] Cunha M., Fernandes B., Covas J.A., Vicente A.A., Hilliou L. (2016). Film blowing of PHBV blends and PHBV-based multilayers for the production of biodegradable packages. J. Appl. Polym. Sci..

[60] Teixeira P.F., Covas J.A., Suarez M.J., Angulo I., Hilliou L. (2020). Film Blowing of PHB-Based Systems for Home Compostable Food Packaging. Int. Polym. Process..

[61] Zambaux M.F., Bonneaux F., Gref R., Maincent P., Dellacherie E., Alonso M.J., Labrude P., Vigneron C. (1998). Influence of experimental parameters on the characteristics of poly(lactic acid) nanoparticles prepared by a double emulsion method. J. Control. Release.

[62] Marcello E., Nigmatullin R., Basnett P., Maqbool M., Prieto A., Knowles J.C., Boccaccini A.R., Roy I. (2024). 3D Melt-Extrusion Printing of Medium Chain Length Polyhydroxyalkanoates and Their Application as Antibiotic-Free Antibacterial Scaffolds for Bone Regeneration. ACS Biomater. Sci. Eng..

[63] Bossu J., Le Moigne N., Dieudonne-George P., Dumazert L., Guillard V., Coussy H.A. (2021). Impact of the processing temperature on the crystallization behavior and mechanical properties of poly [R-3-hydroxybutyrate-co-(R-3-hydroxyvalerate)]. Polym. Int. J. Sci. Technol. Polym..

[64] Bucci D., Tavares L., Sell I. (2005). PHB packaging for the storage of food products. Polym. Test..

[65] Getova Vasilena E., Pascual A., Dijkstra R., Castilla-Casado C., Van Bochove B., Van Osch G.J.V.M., Bernsen M.R., Moreira Teixeira L.S. (2025). Multilayered Tissue Assemblies Through Tuneable Biodegradable Polyhydroxyalkanoate Polymer (Mesh)-Reinforced Organ-Derived Extracellular Matrix Hydrogels. Gels.

[66] Ďurfina M., Babaei N., Vanovčanová Z., Křivská B., Kucharczyk P., Sedlařík V., Hodan J., Kučka J., Sochorová M., Rychtera M. (2025). Bio-Based Polyhydroxyalkanoate (PHA) Blends for 3D Printing: Rheological, Mechanical, Biocompatibility, and Biodegradation Properties. Polymers.

[67] Buyuksoy-Fekraoui K., Mallet K., Benguigui L., Soulestin J., Lacrampe M.-F., Krawczak P. (2022). Characterization of optimized ternary PLA/PHB/organoclay composites processed through fused filament fabrication and injection molding. Materials.

[68] Strangis G., Labardi M., Gallone G., Milazzo M., Capaccioli S., Forli F., Cinelli P., Berrettini S., Seggiani M., Danti S. (2024). 3D Printed Piezoelectric BaTiO3/Polyhydroxybutyrate Nanocomposite Scaffolds for Bone Tissue Engineering. Bioengineering.

[69] Kanabenja W., Passarapark K., Subchokpool T., Nawaaukkaratharnant N., Román A.J., Osswald T.A., Aumnate C., Potiyaraj P. (2022). 3D printing filaments from plasticized Polyhydroxybutyrate/Polylactic acid blends reinforced with hydroxyapatite. Addit. Manuf..

[70] Carvalho D., Gomes J., Zanini N.C., Claro A.M., Amaral D., Cavichiolli N., Barud H.S., Mulinari D.R. (2022). Composite filaments OF PHBV reinforced with ZrO2·nH2O particles for 3D printing. Polym. Bull..

[71] D’arienzo L., Acierno S., Patti A., Di Maio L. (2024). Cellulose/Polyhydroxybutyrate (PHB) Composites as a Sustainable Bio-Based Feedstock to 3D-Printing Applications. Materials.

[72] Şahin G., Özyıldırım H., Şahin A. (2024). Investigation of mechanical and printing properties of poly (lactic acid) and its composite filaments used in 3D printing. Iran. Polym. J..

[73] Dan L., Zhang Y., Zhang L., Liu Y., Li Y., Chen Y. (2021). Three-dimensional printed and biocompatible conductive composites comprised of polyhydroxybutyrate and multiwalled carbon nanotubes. Ind. Eng. Chem. Res..

[74] Zainuddin M.Z., Abu Bakar A.A., Adam A.N., Abdullah S.M., Tamchek N., Alauddin M.S., Mahat M.M., Wiwatcharagoses N., Alforidi A., Ghazali M.I.M. (2023). Mechanical and Structural Properties of Polyhydroxybutyrate as Additive in Blend Material in Additive Manufacturing for Medical Applications. Polymers.

[75] Menčík P., Přikryl R., Krobot Š., Melčová V., Kontárová S., Plavec R., Bočkaj J., Horváth V., Alexy P. (2022). Evaluation of the Properties of PHB Composite Filled with Kaolin Particles for 3D Printing Applications Using the Design of Experiment. Int. J. Mol. Sci..

[76] Moroni S., Khorshid S., Aluigi A., Tiboni M., Casettari L. (2022). Poly(3-hydroxybutyrate): A potential biodegradable excipient for direct 3D printing of pharmaceuticals. Int. J. Pharm..

[77] Pal S., Kridiotis P., Vergel A.J., Medved Z., Barbosa R., Werker A. (2025). Direct melt extrusion of polyhydroxyalkanoate solvent-rich gels after polymer extraction and melt processing with integrated solvent recovery. J. Clean. Prod..

[78] Wei L.Q., Armando G., Nicole MStark M. (2015). Grafting of Bacterial Polyhydroxybutyrate (PHB) onto Cellulose via In Situ Reactive Extrusion with Dicumyl Peroxide. Biomacromolecules.

[79] Aguilar-De-Leyva N., Casas M., Ferrero C., Muñoz-Rubio A., Velasco D., Santoveña B. (2024). 3D Printing Direct Powder Extrusion in the Production of Drug Delivery Systems: State of the Art and Future Perspectives. Pharmaceutics.

[80] Xue J., Xie J., Liu W., Xia Y. (2019). Electrospinning and electrospun nanofibers: Methods, materials, and applications. Chem. Rev..

[81] Raza Z.A., Naeem A.R., Shafi R., Abid S. (2024). Chitosan-incorporated poly(hydroxybutyrate) porous electrospun scaffold for potential biomedical applications. Polym. Bull..

[82] Zhao X., Niu Y., Mi C., Gong H., Yang X., Cheng J., Zhou Z., Liu J., Peng X., Wei D. (2021). Electrospinning nanofibers of microbial polyhydroxyalkanoates for applications in medical tissue engineering. J. Polym. Sci..

[83] Chiesa E., Clerici F., Bucci R., Anastasi F., Bottiglieri M., Patrini M., Genta I., Bittner A.M., Gelmi M.L. (2024). Smart Electrospun Nanofibers from Short Peptidomimetics Based on Pyrrolo-pyrazole Scaffold. Biomacromolecules.

[84] Raza Z.A., Khalil S., Abid S. (2020). Recent progress in development and chemical modification of poly(hydroxybutyrate)-based blends for potential medical applications. Int. J. Biol. Macromol..

[85] Apicella A., Scarfato P., Incarnato L. (2025). Study on 3D printability of PLA/PBAT/PHBV biodegradable blends for packaging applications. Polym. Test..

[86] Yuan H., Tang L., Yang Z.W., Zhang Y., Liu Y., Zhao Z., Li J. (2025). PHB/PBAT blended melt-blown micro-nano fiber carbon source for efficient solid-phase denitrification: Insights into low-carbon treatment and enhanced mechanism. Chem. Eng. J..

[87] Suttiwijitpukdee N., Sato H., Unger M., Ozaki Y. (2012). Effects of Hydrogen Bond Intermolecular Interactions on the Crystal Spherulite of Poly(3-hydroxybutyrate) and Cellulose Acetate Butyrate Blends: Studied by FT-IR and FT-NIR Imaging Spectroscopy. Macromolecules.

[88] Martínez-Sanz M., Villano M., Oliveira C., Albuquerque M.G., Majone M., Reis M., Lopez-Rubio A., Lagaron J.M. (2014). Characterization of polyhydroxyalkanoates synthesized from microbial mixed cultures and of their nanobiocomposites with bacterial cellulose nanowhiskers. New Biotechnol..

[89] Abdalkarim S.Y.H., Yu H.-Y., Wang C., Yang L., Guan Y., Huang L., Yao J. (2018). Sheet-like cellulose nanocrystal-ZnO nanohybrids as multifunctional reinforcing agents in biopolyester composite nanofibers with ultrahigh UV-shielding and antibacterial performances. ACS Appl. Bio Mater..

[90] Wu C.S., Liao H.T., Tsou C.Y., Chen S.C. (2010). Processing and characterization of solid and microcellular PHBV/PBAT blend and its RWF/nanoclay composites. Compos. Part A Appl. Sci. Manuf..

[91] Berthet M.A., Angellier-Coussy H., Chea V., Guillard V., Gastaldi E., Gontard N. (2015). Sustainable food packaging: Valorising wheat straw fibres for tuning PHBV-based composites properties. Compos. Part A Appl. Sci. Manuf..

[92] Asare E., Azimi B., Vasili E., Caporalini S., Azimi B., Zergat S., Ansari Chaharsoughi M., Maleki H., Batoni G., Danti S. (2025). Saverio Caporalini, Bahareh Azimi, Samir Zergat, Mahdi Ansari Chaharsoughi, Homa Maleki, Giovanna Batoni, Serena Danti, Electrospinning Enables Opportunity for Green and Effective Antibacterial Coatings of Medical Devices. J. Funct. Biomater..

[93] Tomano N., Ikuhara T., Hiraishi T., Taguchi S., Kasuya K., Abe H. (2022). Enhancing impact resistance and biodegradability of PHBV by melt blending with ENR. Sci. Rep..

[94] Zhao X., Ji K., Kurt K., Cornish K., Vodovotz Y. (2019). Optimal mechanical properties of biodegradable natural rubber-toughened PHBV bioplastics intended for food packaging applications. Food Packag. Shelf Life.

[95] Jaffur B.N., Kumar G., Khadoo P. (2024). Production and functionalization strategies for superior polyhydroxybutyrate blend performance. Int. J. Biol. Macromol..

[96] Erceg M., Kovacic T., Klaric I. (2005). Thermal degradation of poly(3-hydroxybutyrate) plasticized with acetyl tributyl citrate. Polym. Degrad. Stab..

[97] Wang L., Zhu W., Wang X., Chen X., Chen G.Q., Xu K. (2008). Processability modifications of poly(3-hydroxybutyrate) by plasticizing, blending, and stabilizing. J. Appl. Polym. Sci..

[98] Nosal H., Moser K., Warzała M., Klim M., Zimniewska M., Rydzkowski T., Milewska A., Rydz J. (2021). Selected fatty acids esters as potential PHB-V bioplasticizers: Effect on mechanical properties of the polymer. J. Polym. Environ..

[99] Das S.K., Eshkalak S.K., Chinnappan A., Bhat A.H., AlAliAlMaadeed M.A., Karim A. (2021). Plastic recycling of polyethylene terephthalate (PET) and polyhydroxybutyrate (PHB)-A comprehensive review. Mater. Circ. Econ..

[100] Gong J., Qiang Z., Ren J. (2022). In situ grafting approach for preparing PLA/PHBV degradable blends with improved mechanical properties. Polym. Bull..

[101] Norrrahim M.N.F., Ariffin H., Hassan M.A., Ibrahim N.A., Nishida H. (2013). Performance evaluation and chemical recyclability of a polyethylene/poly(3-hydroxybutyrate-co-3-hydroxyvalerate) blend for sustainable packaging. RSC Adv..

[102] Shahdan D., Rosli N.A., Chen R.S., Ahmad S., Gan S. (2023). Strategies for strengthening toughened poly(lactic acid) blend via natural reinforcement with enhanced biodegradability: A review. Int. J. Biol. Macromol..

[103] Chikh A., Benhamida A., Kaci M., Bourmaud A., Bruzaud S. (2017). Recyclability assessment of poly(3-hydroxybutyrate-co-3-hydroxyvalerate)/poly(butylene succinate) blends: Combined influence of sepiolite and compatibilizer. Polym. Degrad. Stab..

[104] Thanh N.H., Olekhnovich R., Sitnikova V., Kremleva A., Snetkov P., Uspenskaya M. (2023). PHB/PEG Nanofiber Mat Obtained by Electrospinning and Their Performances. Technologies.

[105] Aliotta L., Gigante V., Lazzeri A. (2022). Analytical Modeling of Stress Relaxation and Evaluation of the Activation Volume Variation: Effect of Temperature and Plasticizer Content for Poly (3-hydroxybutyrate-3-hydroxyvalerate). ACS Omega.

[106] Das S.K., Eshkalak S.K., Chinnappan A., Bhat A.H., AlAliAlMaadeed M.A., Karim A. (2022). Atomization of Microfibrillated Cellulose and Its Incorporation into Poly (3-hydroxybutyrate-co-3-hydroxyvalerate) by Reactive Extrusion. Appl. Sci..

[107] Sun S., Liu P., Ji N., Hou H., Dong H. (2017). Effects of low polyhydroxyalkanoate content on the properties of films based on modified starch acquired by extrusion blowing. Food Hydrocoll..

[108] Wu C.S., Liao H.T., Cai Y.X. (2017). Characterisation, biodegradability and application of palm fibre-reinforced polyhydroxyalkanoate composites. Polym. Degrad. Stab..

[109] Zhou T., Wang S., Zhang W., Yin F., Cao Q., Lian T., Dong H. (2023). Polyhydroxyalkanoates production from lactic acid fermentation broth of agricultural waste without extra purification: The effect of concentrations. Environ. Technol. Innov..

[110] Ogura T., Shinzawa H., Nishida M., Kanematsu W. (2016). Tensile properties of polyhydroxyalkanoate/polycaprolactone blends studied by rheo-optical near-infrared (NIR) spectroscopy. J. Mol. Struct..

[111] Sun S., Liu P., Ji N., Hou H., Dong H. (2018). Effects of various cross-linking agents on the physicochemical properties of starch/PHA composite films produced by extrusion blowing. Food Hydrocoll..

[112] Wu C.S. (2018). Characterization, functionality and application of siliceous sponge spicules additive-based manufacturing biopolymer composites. Addit. Manuf..

[113] Mármol G., Gauss C., Fangueiro R. (2020). Potential of Cellulose Microfibers for PHA and PLA Biopolymers Reinforcement. Molecules.

[114] Christian S.J., Billington S.L. (2011). Mechanical response of PHB- and cellulose acetate natural fiber-reinforced composites for construction applications. Compos. Part B Eng..

[115] Gigante V., Cinelli P., Seggiani M., Lazzeri A. (2020). Processing and thermomechanical properties of PHA. The Handbook of Polyhydroxyalkanoates.

[116] Nigmatullin R., Taylor C.S., Basnett P., Pina S., Chaloupka K., Knowles J.C., Roy I. (2023). Medium chain length polyhydroxyalkanoates as potential matrix materials for peripheral nerve regeneration. Regen. Biomater..

[117] Haraźna K., Fricker A.T., Konefał R., Medaj A., Zimowska M., Leszczyński B., Wróbel A., Bojarski A.J., Roy I., Guzik M. (2024). Physicochemical, structural and biological characterisation of poly(3-hydroxyoctanoate) supplemented with diclofenac acid conjugates—Harnessing the potential in the construction of materials for skin regeneration processes. Int. J. Biol. Macromol..

[118] Chuenchart W., Surendra K.C., Khanal S.K. (2024). Understanding Anaerobic Co-digestion of Organic Wastes through Meta-Analysis. ACS ES&T Eng..

[119] Cvek M., Paul U.C., Zia J., Mancini G., Sedlarik V., Athanassiou A. (2022). Biodegradable Films of PLA/PPC and Curcumin as Packaging Materials and Smart Indicators of Food Spoilage. ACS Appl. Mater. Interfaces.

[120] Lee S., Lee I., Seo D., Kim H., Joo G., Lee S., Park K. (2024). Life Cycle Assessment of aPHA Production. ACS Sustain. Chem. Eng..

[121] Yousuf R.G. (2018). Novel Polyhydroxybutyrate (PHB) Production Using a Waste Date Seed Feedstock. Ph.D. Thesis.

[122] Sanchez-Garcia M.D., Gimenez E., Lagaron J.M. (2008). Morphology and barrier properties of nanobiocomposites of poly (3-hydroxybutyrate) and layered silicates. J. Appl. Polym. Sci..

[123] Chen J., Gong C. (2025). Preparation of polyhydroxyalkanoate nanocomposites for biomedical applications. Polym. Int..

[124] Li J., Zhang X., Zhang P. (2025). Polyhydroxyalkanoates (PHAs) as Biomaterials for the Regeneration of Bone Tissue. Polyhydroxyalkanoates: Sustainable Production and Biotechnological Applications III: Biomedical Sector.

[125] Mohamed S.M.D.S., Tuffin J., Watson J., Anderson C., Claeyssens F., Miller C.A., Rook T., Owen R., Irvine S., Locke I.C. (2025). Biosynthesis, characterisation and biocompatibility of a unique and elastomeric medium chain-length polyhydroxyalkanoates for kidney glomerular tissue engineering. Mater. Today Bio.

[126] Maciá Torregrosa M.E., Camacho Diez J. (2025). Shaping a Sustainable Future: Use of Biodegradable Plastics in Ephemeral Constructions//Innovations in Energy Efficient Construction Through Sustainable Materials. IGI Glob..

[127] Jayalath S.U., Alwis A.P.D. (2025). the Greenest Plastic So Far: Advancing Microbial Synthesis. Recovery, and Sustainable Applications for Circularity. ACS Omega.

[128] Jaisri J., Balaji S. (2025). Biodegradable Elegance: Assessing Luxury Textile Biodegradability. Crafting Sustainability in Luxury Textiles for a Zero-Waste Future.

